# Influence of Jackfruit Wood Barrels and Chips During Aging on the Quality and Phenolic Compounds of Cachaça

**DOI:** 10.3390/foods14101812

**Published:** 2025-05-20

**Authors:** Wilton Amaral Santos, Gabriel Benedito Rozendo Bonfim, Jaqueline Santos Jesus, Raimunda Fernandes Souza Fonseca, Maria de Fátima Bomfim da Conceição, Luciane Santos Sousa, Sarah Adriana Rocha Soares, Benjamim Almeida Mendes, Jeancarlo Pereira Anjos, Bruno Martins Dala-Paula, Maria Beatriz A. Gloria, Maria Eugênia Oliveira Mamede

**Affiliations:** 1 Pós-Graduação em Ciência de Alimentos, Universidade Federal da Bahia (UFBA), Salvador 40170-115, BA, Brazil; wiltonasantos1997@gmail.com (W.A.S.); gloria.m@ufba.br (M.B.A.G.); 2 Faculdade de Farmácia, Universidade Federal da Bahia (UFBA), Salvador 40170-115, BA, Brazil; gabrielbomfim58@gmail.com (G.B.R.B.); jaquelinejesus@ufba.br (J.S.J.); rayfernandes20017@gmail.com (R.F.S.F.); anfigarabonfim@gmail.com (M.d.F.B.d.C.); lucianess@ufba.br (L.S.S.); 3 Instituto de Geociências, Universidade Federal da Bahia (UFBA), Salvador 40170-115, BA, Brazil; sarah.ufba@yahoo.com.br; 4 Associação Nacional dos Produtores e Integrantes da Cadeia Produtiva e de Valor da Cachaça de Alambique (ANPAQ), Rua Levindo Lopes, 333, Loja 08, Savassi, Belo Horizonte 30140-170, MG, Brazil; benjaalmendes@gmail.com; 5 Centro de Ciências Naturais e Humanas, CCNH, Universidade Federal do ABC, Santo André 09606-070, SP, Brazil; jeancarlopanjos@gmail.com; 6 Programa de Pós-Graduação em Nutrição e Longevidade, Faculdade de Nutrição, Universidade Federal de Alfenas (UNIFAL-MG), Rua Gabriel Monteiro 700, Alfenas 37130-001, MG, Brazil; bruno.paula@unifal-mg.edu.br

**Keywords:** aeration, innovation, myricetin, copper, zinc, color, piceatannol, ferulic acid, ellagic acid

## Abstract

The traditional aging of cachaça has been performed with different types of wood barrels. Although it is efficient for incorporating phenolics, volatiles, and color into the drink, it is time-consuming for the industry. Alternative aging processes, such as the use of wood chips, have been proposed, and they have the advantage of achieving aging in a shorter time and producing a quality and innovative drink. Therefore, the influence of jackfruit wood barrel and chips and micro-aeration was investigated during cachaça aging. For that, five treatments were used—stainless steel tank (control); stainless steel tank and micro-aeration (control); jackfruit wood barrel; stainless steel tank with jackfruit chips; and stainless steel tank with jackfruit chips and micro-aeration—during two aging times (40 and 79 days). Aging cachaça with jackfruit wood led to increased physicochemical, color, and total phenolic contents, whereas alcoholic degree, lightness, and copper contents decreased. No influence was observed on higher alcohols. Nineteen phenolic compounds were identified in the jackfruit wood aged cachaça by HPLC-DAD-FLD. Myricetin was predominant, a potential marker for jackfruit wood. Longer aging time decreased the alcoholic degree and total ester, but it increased dry extract, density, total and volatile acidity, and it improved the quality of cachaça. The use of chips accelerated aging, whereas micro-aeration led to decreased myricetin. PCA and HCA clustered the samples into three groups: the first was correlated with several flavonoids and coumarins; the second with myricetin, piceatannol, and *trans*-ferulic acid; and the last with ellagic acid. The use of jackfruit chips in the aging of cachaça has been shown to be a promising innovation.

## 1. Introduction

Cachaça is a genuinely Brazilian drink, born in Brazil, and a part of its history [1]. Production techniques have been inherited through several generations, warranting tradition. In addition, it has been a matter of continuous scientific research to improve quality and ensure consumer health [2]. Cachaça is obtained by the distillation, in alembic, of fermented sugar-cane juice with a final alcohol content of 38 to 48% *v/v* at 20 °C [3]. In Brazil, it is the most consumed distilled beverage. In 2022, its exportation market generated USD 20.80 million, an increase of 52.4% compared to 2021, and an additional 0.7% increase in 2023 compared to 2022. In 2022, cachaça became the world’s third most consumed spirit, behind only vodka and the Korean spirit soju [4]. The Brazilian scenario is very encouraging for cachaça, and the state of Minas Gerais stands out for the best quality cachaças and largest production. Cachaça is currently one of the products that generate the most jobs and income in the state.

Like some other alcoholic beverages, cachaça can be aged or not. Aging is a process that brings sensorial changes that consumers appreciate, making it a valued process in the sector [5]. During aging, flaws or aggressive notes in the beverage are mitigated, with a consequent improvement in flavor [6,7]. In the traditional aging process, cachaça stays in a wooden barrel for two years, so that phenolic compounds are transferred from the wood to the drink [3,8]. During this period, wood components, such as volatile oils, phenolic compounds, sugars, non-volatile organic acids, and tannins, among others, are extracted into the beverage, at a certain quantity and quality, depending on the contact [7,9,10]. Several chemical reactions can occur, contributing to the formation of aldehydes and their corresponding acids, including the formation of volatile esters, impacting the quality of the cachaça [7]. In addition, oxidation reactions can occur concomitantly, as oxygen permeates the beverage through the space between barrel staves [1]. Other characteristics that draw attention to aged cachaças are the color due to the migration of phenolic compounds from the wood to the beverage, making it yellowish and more attractive to consumers [3]. Another possibility is that some of the wood compounds could contribute to health-promoting properties [11,12].

Even though aging can bring several benefits to cachaça quality, the traditional process can take up to 24 months to produce the drink. In this long period, losses may occur due to the evaporation of alcohol [3], leading to higher costs and the need for a larger storage capacity [13]. To accelerate the aging of alcoholic beverages, alternative processes have been investigated, such as the use of wood chips [1,13] and forced oxygenation [14,15,16]. Recently, Brazilian legislation allowed the use of wood fragments—wood chips in the aging process [3]. Its use during cachaça aging can lower costs compared to aging in the barrel. In addition, forced oxygenation of the beverage, for example, forced micro-oxygenation, combined with wood chips, can accelerate the aging process [14,16,17,18]. Besides the high-quality drinks obtained, a more sustainable aging process has been associated with the combination of these techniques, which is a remarkable aspect [1,6,17].

Among the most widely used woods to make aging barrels, oak stands out as it is also used for whiskey and wine. However, oak is native to the northern hemisphere, making its use difficult and expensive in Brazil [9]. On the other hand, Brazil is rich in many other types of wood that could be used, imparting interesting characteristics to aged cachaça. Brazilian legislation authorizes the use of alternative wood species in the manufacture of barrels for cachaça aging, including peanut, araruva, cabreúva, cherry, grápia, and purple ipê, among others [5]. Each wood is unique considering its chemical composition, density, and tannin content, among other factors, and, therefore, each one can contribute differently to the beverage [19].

The jackfruit (*Artocarpus heterophyllus*) tree has a widespread distribution in different regions of the Brazilian Atlantic Forest biome, especially the properties of small landholder farmers. It has a great capacity to grow and produce seeds in various climatic and soil conditions [20]. As it is widely available, its use in cachaça aging would be inexpensive and would not be of ecological concern. However, studies are needed to investigate its impact on the chemical and color characteristics of the cachaça and compliance with standards of quality.

The use of wood chips during cachaça aging and micro-aeration are promising innovations towards beverage quality, and that jackfruit wood, although widespread, is scarcely investigated for this purpose, the objective of this study was to investigate the influence of jackfruit wood chips and barrels during cachaça aging, its impact on the physicochemical characteristics, and the migration of phenolic compounds. In addition, the influence of forced micro-aeration was investigated. It is believed that these alternatives can have positive impacts on the quality of the drink, contributing to innovative and more sustainable cachaça production.

## 2. Materials and Methods

### 2.1. Samples and Reagents

Cachaça (same batch, 2019 harvest), with an alcohol content of 48.3% (*v*/*v*) at 20 °C, was kindly provided by Cachaçaria Santíssima (Pitangui, MG, Brazil). Ultra-pure water (18.2 MΩ cm resistivity at 25 °C, total organic carbon *<* 10 μg/L) was obtained from the Direct-Q3^®^ Millipore system (Burlington, MA, USA). All reagents were analytical grade, whereas the solvents for the chromatographic analysis were HPLC and GC grades. The 1-butanol, 2-butanol, methanol, isoamyl alcohol, isobutyl alcohol, and isopropyl alcohol were from Merck (Darmstadt, Germany). The standards of phenolic compounds were from different sources: kaempferol, kaempferide, *trans*-ferulic acid (Fluka, Honeywell International Inc., Charlotte, NC, USA), coumarin (Greentec Soluções & Reagentes, Diadema, SP, Brazil), 7-hydroxycoumarin (Acros Organics, Thermo Fisher Scientific Inc., Waltham, MA, USA), biochanin A, *trans*-cinnamic acid, caffeic acid, catechin, *p*-coumaric acid, ellagic acid, gallic acid, rutin, myricetin, quercetin, *iso*liquiritigenin, naringerin, piceatannol, resveratrol, 7-hydroxycoumarin, scopoletin, and 4-methyl-umbelliferone (Sigma Chemical Co., St. Louis, MO, USA).

### 2.2. Experimental Design

**Figure 1:**
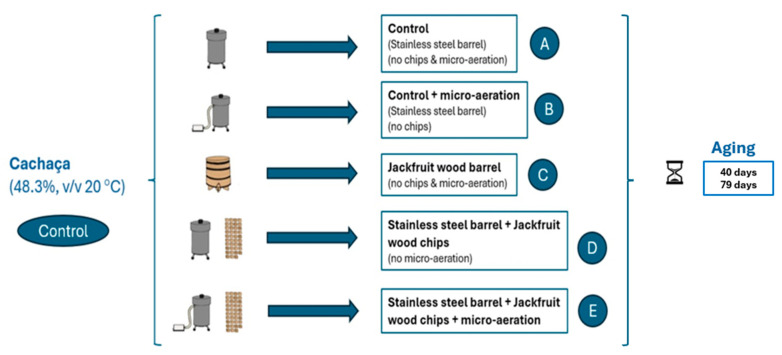
Experimental design of the aging of cachaça in jackfruit wood (barrel or chip) under micro-aeration or not. **Control:** non-aged cachaça; **A**: stainless steel tank (control); **B**: stainless steel tank and micro-aeration (control); **C**: jackfruit barrel; **D**: stainless steel tank with jackfruit chips; **E**: stainless steel tank with jackfruit chips and micro-aeration. Aging time: 40 and 79 days.

The cachaça was homogenized and divided into five different aliquots (7 L) into three repetitions to conduct the studies, plus a Control (non-aged cachaça), totaling 105 L. Each aliquot was subjected to a specific experimental condition (Figure 1), including the following: (A) control treatment—cachaça in a stainless steel tank; (B) control treatment with micro-aeration—cachaça in a stainless steel tank with micro-aeration; (C) cachaça stored in a jackfruit barrel; (D) cachaça stored in a stainless steel tank, with jackfruit chips; and (E) cachaça stored in a stainless steel tank, with jackfruit chips, and micro-aeration. All the treatments were kept in an odor-free environment under controlled temperature and relative humidity (25 ± 3 °C and 60%, respectively). After 40 and 79 days, samples were taken and analyzed. This time was estimated based on calculations described by Ratkovich et al. [1]. The original cachaça (not aged) was analyzed initially and used as a control.

The wooden barrels (*n* = 3) were made of jackfruit (*Artocarpus heterophyllus*) wood and were kindly provided by Tanoaria Dornas Havanas (Taiboeiras, MG, Brazil). The barrels had a 7 L capacity and internal dimensions of 28.5 cm in height and 16.7, 18.7, and 17.7 cm internal diameter at the lid, bottom, and middle height, respectively. The volume of wood used in the wet section was 6233 dm^3^, and the useful specific surface (internal area/internal volume) of the barrel was 2.962 cm^2^/dm^3^ [21]. The thickness of the staves was 3 cm. Thirty-eight units of jackfruit wood chips (polyhedral shape) were used in contact with the cachaça to provide a wetted surface of 20,776 dm^2^, equal to that of the wooden barrels. The stainless steel tanks (a total of 12) were kindly provided by Alambiques Santa Efigênia (Itaverava, MG, Brazil). They were made of AISI 305 stainless steel, with a useful 10 L volume (27 cm height, 21 cm diameter, and a projection of 1 to 2 cm), containing a system that prevented liquid evaporation and losses. Each tank contained a micro-aeration device, consisting of an air compressor (Boyu Sc-7500, Sorocaba, SP, Brazil), and a porous plate, which enabled the formation of microbubbles at a flow rate of 3 L/min (0.429 vvm—air volume/medium volume/min), injecting an average of 19.506 g of air/day. Micro-aeration was carried out twice a day, at intervals of 12 h, lasting 30 min each.

At the beginning of the study (time 0) and at 40 and 79 days of storage, samples were collected and stored in amber bottles, under refrigeration, until analysis for alcohol content, density, total and volatile acidity, total esters, dry extract, total sugars, methanol, higher alcohols, total phenolic compounds, color characteristics, and inorganic contaminants.

### 2.3. Physicochemical Characterization

The samples were analyzed for quality parameters [22], including alcohol content, density, total and volatile acidity, total esters, dry extract, methanol, and total sugars. For the analysis of alcohol content, total esters, total and volatile acidity, methanol, butanol, and higher alcohols, an electronic wine distiller (super DEE DensiMat, Gibertini Elettronica™, Milan, Italy) was used. The alcohol content (% *v*/*v*) and the density were determined using an electronic hydrostatic balance (Super Alcomat, Gibertini Elettronica™, Milan, Italy). The volatile acidity was determined by vapor entrainment of volatile acids in an Enochimic Electronic Distiller, followed by titration with NaOH (0.1 N). Total acidity was determined by titrating the pure beverage with NaOH (0.5 N) using a phenolphthalein indicator. Total esters were determined by titration of the carboxylic acids obtained from the transesterification of the esters present in the samples, and the results were expressed as mg of ethyl acetate/100 mL of anhydrous alcohol. The dry extract was determined by gravimetry. The remaining solid residue was weighed, and the results were expressed in g of dry extract/L of the sample. Total sugar was determined by gravimetric titration using the Fehling method, and the results were expressed in g sucrose/L sample.

The analyses of the higher alcohols isopentyl (isoamyl), isobutyl, propyl, n-butyl, 2-butyl, and methyl were carried out by gas chromatography with a flame ionization detector (GC-FID), according to Barbosa et al. [2]. The redistilled aliquots of the samples were used. The GC used was a Clarus 580 (PerkinElmer, Waltham, MA, USA), with a DB Wax column (30 m × 0.25 mm; 0.25 μm). The carrier gas was nitrogen at 1.4 mL/min. The injector and detector temperatures were 150 and 170 °C, respectively. The volume injected was 1.0 µL in the split mode (1:10). The chromatographic run was set at 35 °C for 4 min, ramp to 80 °C at 10 °C/min, held for 1 min, ramp to 120 °C at 20 °C/min, held for 1 min, ramp to 140 °C at 25 °C/min, and held for 30 s, for a total of 13.48 min. The identification was possible by comparing retention times with standards, and the quantification of analytes was performed by interpolation in external analytical curves. The results were expressed in mg/100 mL of anhydrous alcohol. The samples were also analyzed for total phenolic contents according to the Folin–Ciocalteu method [23], and the results were reported in mg gallic acid equivalents (GAE) per L.

### 2.4. CIE L*a*b* Color Characteristics

The color characteristics of the cachaças resulting from the CIE L*a*b* system, coupled with a Konica Minolta CR-5 colorimeter (Tokyo, Japan), in which L* represents lightness (0% for dark/fully opaque to 100% for bright/fully transparent), and a* and b* are two color ranges from green to red (*x*-axis) and blue to yellow (*y*-axis), respectively, with values ranging from −60 to +60. Based on the values of L* (lightness), a* (variation between green-negative and red-positive), and b* (variation between blue-negative and yellow-positive), the saturation (C) and hue (h) were calculated. A D65° illuminant and a 10° observation angle were used, as well as a 2 mm thick cuvette with a volume of 2 mL, according to Saito et al. [24].

### 2.5. Inorganic Contaminants

The analysis of the inorganic contaminants copper, lead, aluminum, cadmium, and zinc was undertaken by Flame Atomic Absorption Spectrometry (FS 220 FAAS, Varian, Mulgrave, VIC, Australia) as described by Rodrigues et al. [9]. The air/acetylene flame was used for all elements, except for Al, for which the nitrous oxide/acetylene flame was used. Detection was performed at 309.3, 324.8, 213.3, 228.8, and 217.0 nm for Al, Cu, Zn, Cd, and Pb, respectively. The analytical curves were constructed with standard solutions of the elements in the ranges, in mg/L, of 0.25 to 2.00 (Al), 0.20 to 2.00 (Cu), 0.10 to 1.0 (Zn), 0.010 to 0.10 (Cd), and 0.20 to 2.0 (Pb).

### 2.6. Analysis of Phenolic Compounds by HPLC

Nineteen phenolic compounds were quantified by high-performance liquid chromatography with diode array and fluorescence detectors (HPLC-DAD-FLD) in series (Shimadzu Corp., Kyoto, Japan) as described by Lima et al. [25]. Before HPLC analysis, the samples were filtered through 0.45 μm regenerated cellulose membranes (Merck, Darmstadt, Germany). The chromatographic analysis was performed using a Nucleodur^®^ 100–5 C18 (150 × 4 mm, 5 μm) column (Macherey-Nagel, Düren, Germany) coupled to a Zorbax Eclipse Plus C18 (4.6 × 12.5 mm) pre-column (Agilent Technologies, Santa Clara, CA, USA) at 40 °C. The mobile phases A (5% acetic acid) and B (70% ethanol) were used in a gradient (0–22.5 min: 0 to 59% B; 22.5–24.0 min: 59 to 0% B; and 24.0–26.0 min: 0% B) at 1.0 mL/min. The injection volume was 20 μL. Each component was quantified at a specific wavelength [25]. The DAD wavelengths were 260 nm (biochanin A), 270 nm (gallic acid), 280 nm (catechin, coumarin, *trans*-cinnamic acid), 290 nm (naringerin), 310 nm (*p*-coumaric acid), 323 nm (caffeic acid), 354 nm (rutin), 365 nm (kaempferol and kaempferide), 367 nm (ellagic acid), 370 nm (quercetin and *iso*-liquiritigenin), and 372 nm (myricetin); and the excitation/emission wavelengths for the FLD were 280/440 nm (7-hydroxycoumarin, scopoletin, *trans*-ferulic acid, and 4-methyl-umbelliferone) and 330/400 nm (piceatannol and resveratrol). The quantification of compounds was carried out by interpolation in external analytical curves (R^2^ ≥ 0.9901).

### 2.7. Statistical Analysis

The experimental results were evaluated by analysis of variance (ANOVA), and the means were compared by the Tukey test (*p* = 0.05) using the XLSTAT^®^ software (version 2022.4.5, 2023).

Two multivariate exploratory techniques, Principal Component Analysis (PCA) and Hierarchical Cluster Analysis (HCA), were used to characterize the cachaça samples regarding physicochemical parameters, inorganic compounds, alcohol profile, and total levels, total phenolic contents, and CIE L*a*b* color characteristics of the samples from all the different treatments (Control, A to E). PCA and HCA were also used to characterize the treatments in which jackfruit wood (C, D, and E) was used, considering only the phenolic compounds. In PCA, all results were used as active variables in the derivation of the principal components. PCA was performed using the correlation matrix. The dendrogram for HCA was obtained by clustering the same variables used for PCA. Average linkage by squared Pearson was applied to determine the distance between observations and phenolic compounds (Minitab^®^, v. 16.2.3).

## 3. Results and Discussion

### 3.1. Impact of the Treatments on the Physicochemical Characteristics

The physicochemical characteristics of the cachaça subjected to the different treatments are indicated in Table 1. There was a significant difference between treatments (Control, A to E) and between aging times (40 and 79 days). Nonetheless, the samples from every treatment complied with the legislation [3] for the alcoholic degree (38–48%), volatile acidity (<150 mg acetic acid/100 mL), and total ester (<200 mg ethyl acetate/100 mL anhydrous alcohol).

During aging, there were significant decreases in the alcoholic degree (*p* < 0.05), whereas the other parameters increased significantly. These results are consistent with reports from the literature [21,26]. Several factors can contribute to the decrease in the alcoholic degree of cachaça during aging, including the esterification of ethanol with acids [1]. In addition, there can be a loss of volatile alcohols through the wood’s pores [2], an average of 3 to 4%. When using the jackfruit barrel (treatment C), there was a 5.9% decrease. However, according to Morais et al. [27], the type of wood can affect the rate of alcoholic degree reduction, due to the porosity of the wood. They found 4.0 to 9.4% losses when using sassafras (*Ocotea* sp.) and jatobá (*Hymenaea* sp.), respectively.

The alcoholic degree was lower for the E and B treatments (11% and 7% lower, respectively), e.g., treatments in which micro-aeration was used. This result suggests that aging acceleration by aeration can lead to the evaporation of alcohol. In addition, at longer aging times (B—79 and E—79), there were lower alcoholic degrees (4.9% and 3.9% losses, respectively) compared to the respective treatments at 40 days of aging. Therefore, the decrease in the alcoholic degree can result from the esterification reactions, losses through the wood, and aeration [27].

There was a higher density in all the treatments compared to the Control, with higher values observed for treatment E (jackfruit chips and micro-aeration). Therefore, jackfruit wood (barrel or chips) and micro-aeration led to higher density. In addition, the longer the aging time, the higher the density of the cachaça.

The total acidity values varied significantly with changes affected by the treatment. The use of jackfruit wood led to higher total acidity, with higher levels when chips were used (treatment D—40)—96.88 mg acetic acid/100 mL—compared to the barrel (treatment C—40)—68.63 mg acetic acid/100 mL. Therefore, jackfruit wood chips aging led to higher acidity than the barrel. Micro-aeration did not increase acidity, as there were no significant differences between treatments B and A and E and D (in 40 days). However, the longer the aging time in the presence of jackfruit wood (barrel or chip), the higher the total acidity. The increase in acidity can result from the migration of acidic compounds from the wood and from oxidation reactions.

During aging and storage, the oxidation of ethanol into acetaldehyde and the consequent formation of acetic acid can occur, as well as the extraction of organic compounds from the wood that will contribute to the acidity [28]. Small amounts of acids in sugar cane spirits are desirable, as these substances can react with the alcohols, producing esters, which are important for the sensorial characteristics of cachaça [29].

Higher total and volatile acidities were observed when jackfruit wood, both barrels and chips, was used. The longer the aging time, the higher the total acidity (C—79, D—79, E—79). However, aging time increased volatile acidity only when aeration was used (E—79 × E—40). Therefore, the acids extracted from jackfruit wood and those produced during aging were not volatile, which is a positive finding, since volatile acidity is limited by legislation [28]. The volatile acidity found for jackfruit wood aging is like that reported for oak, and lower than amburana (*Amburana cearenses*), at 50 and 300 mg acetic acid/100 mL anhydrous alcohol, respectively [29].

Higher total ester contents were observed in the treatments with jackfruit wood chips, without (D—40) or with aeration (E—40). And the levels decreased as aging time increased from 40 to 79 days. Small quantities of esters in cachaça are desirable, as these substances are responsible for the characteristic flavor and aroma [29]. However, they are undesirable at high concentrations [7], as they are limited by legislation—200.0 mg/100 mL of anhydrous alcohol [3]. Esters are mainly formed during the fermentation of sugar cane through the metabolism of *Saccharomyces cerevisiae*, which is the reason why the fermentation must be well controlled [30]. The formation of esters during aging in the presence of jackfruit wood reflects the esterification of acids that migrated from the wood [2], contributing to the cachaça’s bouquet.

The dry extract of the cachaças was significantly higher for the treatments in which jackfruit wood was used (C, D, and E) compared to the controls (Control, A, and B). When using the chips of jackfruit wood (treatments D and E), higher dry extract contents were found compared to the use of the jackfruit wood barrel (treatment C), but they did not differ statistically. Longer aging times only increased the dry extract when aeration was used (treatment E—79). The increase in dry extracts occurs as some tannins and phenolics from the degradation of the wood lignin migrate into the drink [1], which is why it can be a marker of wood aging. Dry extracts up to 10.81 g/L were obtained, showing an efficient migration of compounds from the wood to the beverage. This value is higher than the 0.06–0.19 g/L and 0.30–1.22 g/L found in cachaças aged in oak and amburana barrels for 12 months [29].

Jackfruit chips (treatments D and E) led to higher total sugar levels compared to the use of barrels (treatment C). Aeration and increased aging time did not affect total sugar. Jackfruit chips (treatments D and E) provided a 3.3-fold increase in total sugar. Sugar, even in small amounts, can improve the palatability of cachaça. According to Zacaroni et al. [31], the increase in sugar contents in alcoholic beverages aged in wood can result from the breakdown of hemicellulose and its derivatives.

### 3.2. Impact of the Treatments on Inorganic Contaminants

Among the five inorganic contaminants analyzed, only copper and zinc were detected in the samples (Table 1). Lead, aluminum, and cadmium were not detected in any sample. Copper was present in every sample at levels ranging from 0.53 to 1.23 mg/L, whereas zinc was only detected in the control treatments without jackfruit wood (A and B) at levels from 0.11 to 0.16 mg/L. The presence of copper in spirits is mainly due to the distillation process in stills constructed with this metal. Copper contamination can increase when the cleaning of the distillation equipment is inadequate [1]. Copper, at high levels, can cause adverse effects on human health [1,31], and it can catalyze the formation of carcinogenic ethyl carbamate when present in high concentrations [1]. Therefore, Brazilian legislation establishes a maximum of 5 mg/L of copper in cachaça [3], and other countries have a stricter limit of 2 mg/L [31]. In all five treatments, the levels of copper complied with Brazilian and international legislations. The levels of copper in the treatments in which jackfruit barrels or chips were used (C, D, and E) were lower (*p* < 0.05) compared to the control and other treatments (A and B), and the changes were not affected by the aging time. The loss of copper during aging in wood barrels can result from the absorption of copper ions by the wood [32], especially by its fibers [1,2]. In addition, copper chemical and redox speciation can play a role in its stability in alcoholic beverages [33], which deserves further studies. Therefore, besides providing desirable flavor and color characteristics, wood aging can provide benefits regarding the lower copper contents in the beverage.

The levels of zinc were ≤0.16 mg/L and it was not detected in the samples aged with jackfruit wood, either barrel or chips. These levels complied with general food legislation [34], whereas none is available for cachaça [3]. This result suggests that zinc is also absorbed by wood, as reported for copper [32]. Aging time, from 40 to 79 days, did not affect the levels of Zn in the treatments. Even though aluminum is the most abundant metal in the lithosphere [35], it was not detected in any samples (<0.20 mg/L). The levels of cadmium and lead were also below the quantification limits, and, consequently, below 0.02 mg/L and 0.1 mg/L, respectively. These results indicate that the combination of stainless steel barrel and jackfruit wood in the aging of cachaça does not present a risk of contamination of the drink by heavy metals; therefore, it does not present a risk to the consumer.

### 3.3. Impact of the Treatments on Higher and Butyl and Methyl Alcohols

The results for the higher butyl and methyl alcohols in the *cachaça* following the different treatments are described in Table 2. After ethyl alcohol, higher alcohols are the most predominant volatile compounds in *cachaça*. These compounds can contribute to the characteristic flavor and aroma [36]; however, when at higher levels, they can negatively affect these characteristics [1]. According to Brazilian legislation [3], the sum of propyl, isobutyl, and isoamyl alcohols must not exceed 360 mg/100 mL of anhydrous alcohol. The levels found in the samples were much lower (<28.2 mg/100 mL of anhydrous alcohol). And there was no significant difference between treatments. Higher alcohols can be produced by the deamination of amino acids under anaerobic conditions and by the decarboxylation of sugars under aerobic conditions during fermentation [36], and due to water evaporation and some changes in the fractions of higher alcohols during aging [16]. So, the use of jackfruit wood (barrel or chip), micro-aeration, and an aging time of up to 79 days did not affect the alcohol levels of the cachaça.

Isobutyl, isoamyl, and propyl alcohols are also described in Table 2. The levels of isobutyl alcohol were lower compared to the control (5.62 mg/100 mL anhydrous alcohol) in most of the treatments, except for treatment E (aging in the presence of jackfruit wood chips and micro-aeration), which did not differ significantly from the control. No significant difference (*p* > 0.05) was observed for the isopentyl alcohol among treatments with levels around 9 mg/100 mL of anhydrous alcohol.

The presence of n-butyl, 2-butyl, and methyl alcohol was not observed in any sample. According to Brasil [22], 1- and 2-butyl alcohols are usually contaminants, even though these alcohols can be formed during fermentation, often associated with bacterial contamination [1]. These compounds were not detected (quantification limit = below 0.36 and 0.48 mg/100 mL anhydrous alcohol, respectively) in any of the samples, and, therefore, they complied with Brazilian legislation (3 and 10 mg/100 mL, respectively) [22]. Methyl alcohol can be found in alcoholic beverages, due to the degradation of pectin during fermentation [36]. The presence of methyl alcohol is undesirable since this substance can cause adverse effects on human health, including blindness and death [37,38]. Table 2 shows the levels of some alcohols and total higher alcohols in *cachaça* submitted to different treatments—not aged (Control), aged in stainless steel or jackfruit barrels in the presence or not of jackfruit wood chips, and micro-aerated or not (A–E) for 40 and 79 days.

The concentrations of methyl alcohol for all the treatments were below the detection limit of the method (<0.19 mg/100 mL anhydrous alcohol), which complies with Brazilian legislation—20.0 mg/100 mL anhydrous alcohol [3]. This is a promising result when compared to other studies in which this compound was found at 5.19 to 5.76 mg/100 mL in aged cachaças from the market [13,27,29].

### 3.4. Impact of the Treatments on the CIE L*a*b* Color Characteristics

The CIE Lab characteristics of the samples are described in Table 3. As expected, the lightness (L*) was affected using jackfruit wood, with lower values (*p* < 0.05) for chips compared to the barrel. Micro-aeration and aging time did not significantly affect lightness (L); a* values were negative (greenish) for Control, A, and B treatments, and no significant difference was observed among them. With the use of jackfruit wood, a* values became positive (reddish), and the values for the treatments with the chips led to higher a* values than the use of barrels. Aging time and micro-aeration did not affect a* values (*p* > 0.05). Similarly, the use of jackfruit wood increased b* values, and no significant difference was observed for barrels or chips and micro-aeration. The longer aging caused an increase in b* values only for treatment C, in which a jackfruit barrel was used. These changes are better understood when combining the CIE parameters and comparing chroma and hue values. The use of wood affected the color, leading to higher chroma and lower hue, and these changes were not affected by using barrels or chips, by micro-aeration, and by the longer aging times (79 compared to 40 days aging). This result suggests that the greatest color change occurred in the first 40 days of aging, irrespective of the wood contact (chip or barrel), remaining stable for up to 79 days. The transfer of pigments and color characteristics of jackfruit to wine was also observed during wine aging in a wood barrel [12].

### 3.5. Total Phenolic Contents

The total phenolic content determined by the Folin–Ciocalteu method was only detected in treatments with jackfruit wood, e.g., treatments C, D, and E (Table 4). Phenolic compounds can migrate from the wood into the cachaça, and thereby, they can affect the quality of the beverage. In addition, it can be used to monitor the wood aging process [3,39]. When comparing the treatments, higher total phenolic contents were observed when chips were used, compared to the barrel, but without a significant difference. Micro-aeration did not affect total phenolics. The longer aging time (from 40 to 79 days) caused a further significant increase in the total phenolic content.

### 3.6. Multivariate Analysis of Physicochemical and Color Parameters

**Figure 2:**
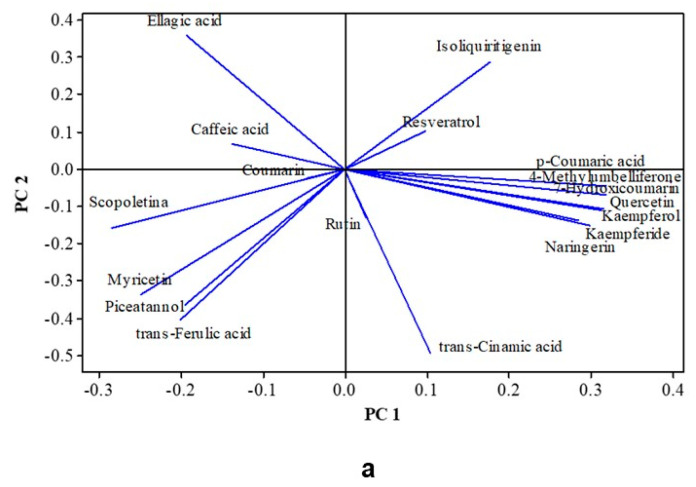

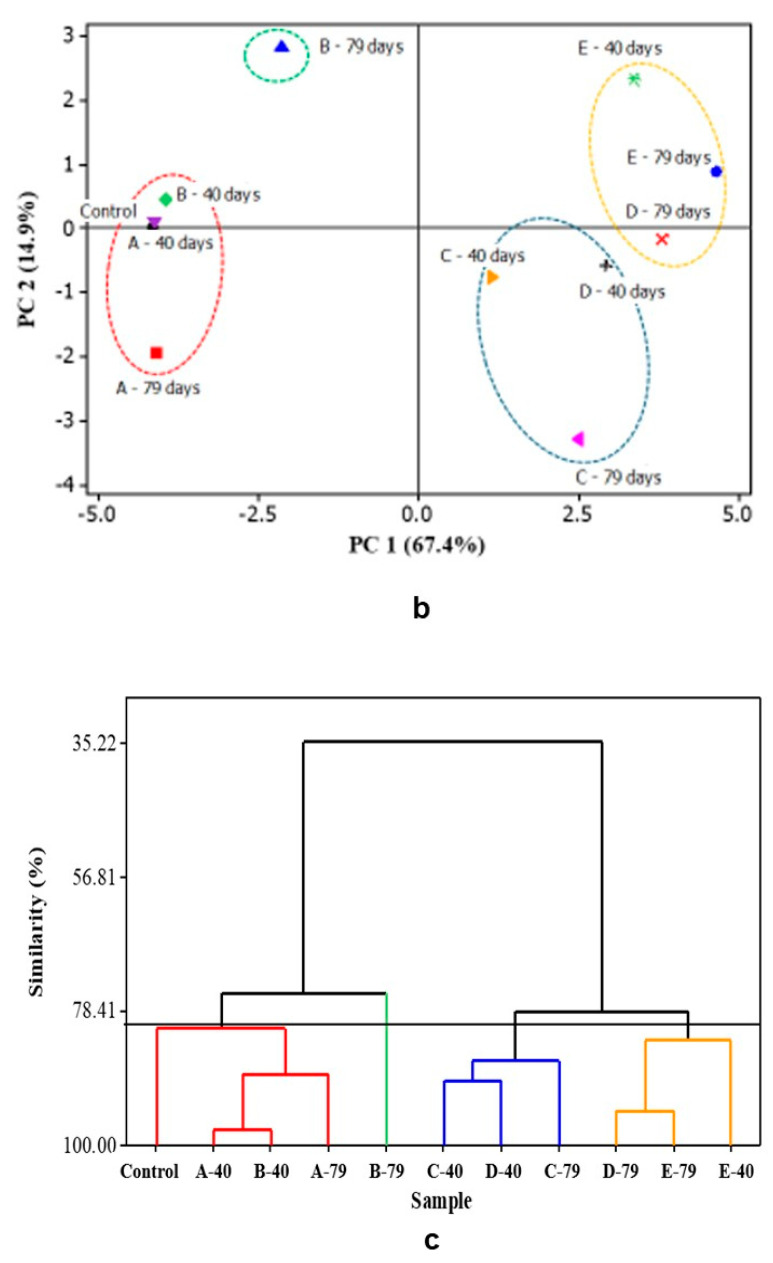
Principal Component Analyses (PCAs) and Hierarchical Cluster Analyses (HCAs) of physicochemical parameters and CIE L*a*b* color characteristics of cachaça aged or not (control) in jackfruit wood barrel or chip, under micro-aeration or not. (**a**) Loadings plot (PC1 + PC2 = 82.3%); (**b**) score plot showing clusters according to 80% similarity HCA; and (**c**) dendrogram obtained by clustering individual values using average linkage and squared Pearson distance. Parameters: alcoholic degree (%*v/v*), density at 20 °C (g/L), total acidity (mg acetic acid), volatile acidity, total esters, dry extract (g/L), total sugars (g/L), ∑ higher alcohols (isobutyl alcohol, isoamyl alcohol, propyl alcohol), phenolics compounds (mg/L), Ash, Cu (mg/L), Zn (mg/L), and color L*, a*, b*, c*, h. Each colored group (circle) in (**b**) corresponds to a cluster in (**c**).

Multivariate analyses of the auto-scaled data indicated that a two-principal components (PC) model explained 82.3% of the variance: 67.4% by PC1 and 14.9% by PC2 (Figure 2). According to the PC loadings (Figure 2a) and the positions of the samples in the quadrants (Figure 2b), it is possible to confirm that PC1 separated the treatments in which jackfruit wood was used (positive values)—treatments C, D, and E—from the treatments in which jackfruit wood was not used (treatments A and B) and from Control (negative values), irrespective of the aging time. The most significant positive effects on PC1 were a*, b*, and c* values, the levels of total and volatile acidity, total sugars, and total phenolic contents, whereas L*, h, zinc, and copper were affected negatively. PC2 separated B-79 from the other treatments that did not use wood (A, B, and Control) due to the levels of isoamyl alcohol and ∑higher alcohols, suggesting that micro-aeration of cachaça could negatively affect these parameters. PC2 also separated the treatments using jackfruit barrels and chips based on isoamyl alcohol and ∑higher alcohols, which were higher in D—79, E—40, and E—79, compared to the other wood-aged samples and lower alcoholic degree.

HCA (Figure 2c) clustered, with ~80% similarity, the same four groups from PCA, e.g., the samples that were not in contact with the jackfruit wood clustered in one group with Control, A—40, A—79, and B—40, and a second one only with B—79. The treatments with jackfruit wood were also separated into two clusters. The first included treatments E—40, E—79, and D—79, and the second was with treatments C—40, C—79, and D—40.

### 3.7. Impact of the Treatments on Some Specific Phenolic Compounds and Coumarins

The aging of cachaça in wood barrels is becoming common among producers as it can add value to the product. During this period, several compounds from the wood are incorporated into the beverage, providing attractive flavors and colors [21]. When considering the individual phenolic compounds and coumarins determined by HPLC, 19 compounds (Table 4) were quantified. These compounds were only present in samples from the treatments in contact with jackfruit wood (C, D, and E) and probably migrated from it. Some compounds were present at low levels, near the limit of quantification of the method, e.g., scopoletin (≤0.47 mg/L), which contributed only 1.25% of the total level. Eight compounds (coumarin, *trans*-cinnamic acid, ellagic acid, rutin, kaempferol, piceatannol, resveratrol, and *trans*-ferulic acid) had similar levels for all the treatments, suggesting that their extraction from the wood was not affected either by the type of contact (barrel or chips) or by micro-aeration. Other compounds, including naringerin, 7-hydroxycoumarin, 4-methyl-umbelliferone, *p*-coumaric acid, and quercetin, were significantly higher in treatment E—79. This result suggests that contact with the jackfruit wood chips under micro-aeration at longer times favored the formation and buildup of these compounds. Kaempferide was found at higher levels in E—40 and E—79, and no significant difference was observed between them, e.g., it was not affected by aging time. This compound was enhanced using wood chips and micro-aeration. Caffeic acid was present at higher levels in E—40, the treatment in which wood chips were used under micro-aeration; however, at longer aging time (79 days), the levels were lower, probably due to the degradation of this compound with time. Myricetin was found at significantly higher levels in treatments D—41 compared to C—40, C—79, E—40, and E—79. This result suggests that myricetin was most effectively extracted from the jackfruit chips, compared to the use of jackfruit barrels and micro-aeration. However, when using a longer exposure time to chips (D—79), no significant difference was observed from both C and E, suggesting that there could be a loss of myricetin as exposure time increases. In fact, according to the literature [40], myricetin strongly scavenges free radicals; therefore, it is oxidized, protecting other components in the aged cachaça. For *iso*-liquiritigenin, significantly higher levels were found in treatments C—79 and E—79, e.g., those involving the use of micro-aeration for a longer time.

When considering the contribution of each compound to the total level, in C—40 and D—40, myricetin contributed the most (24.6 and 38.2%, respectively) to total levels, followed by *trans*-ferulic acid (15.4 and 25.2%, respectively) and caffeic acid (13.0 and 8.2%, respectively). In D-79, myricetin was prevalent (33.3%), followed by rutin (11.7%), and caffeic acid (7.0%). The predominance for E-40 was different, e.g., caffeic acid (26.4%), followed by myricetin (23.4%) and *trans*-ferulic acid (11.4%). In the treatment E-79, the predominant was *p*-coumaric acid (21.8%), followed by quercetin (17%) and 4-methyl-umbelliferone (11.2%). Overall, a balance between additive and subtractive phenomena during the aging process was observed, which was affected by the specific conditions of each treatment.

Most of these predominant compounds from jackfruit wood are plant secondary metabolites with beneficial effects on health [41,42,43]. Myricetin shows anticancer, antidiabetic, and antiobesity factors. It provides cardiovascular, osteoporosis, anti-inflammatory, and hepato protection [43]. It has also been reported to protect against Parkinson’s and Alzheimer’s diseases [42]. It was found to be non-toxic towards normal cells [42]. *Trans*-ferulic acid has many pharmacological properties, such as antioxidant, antimicrobial, antifungal, and anti-inflammation [44]. In addition, it was reported to have an anxiolytic activity [45]. Caffeic acid shows antimicrobial and antioxidant properties [46] and prevents inflammation, cancer, neurodegenerative diseases, and diabetes [47]. Ellagic acid has anti-mycobacterial, anti-inflammatory, anti-hyperlipidemic, and neuroprotective activities and the potential for treating breast cancer and gut inflammation [48]. In addition to its antioxidant capacity, quercetin has carcinostatic and antiviral properties. It is antihypertensive and anti-inflammatory, offers protection of low-density lipoprotein (LDL) from oxidation, and the inhibition of angiogenesis [49]. At last, 4-methyl-umbelliferone has shown choleretic and antispasmodic properties with the potential to prevent biliary spasms [50]. The other compounds also have interesting health benefits, including piceatannol and resveratrol [51,52].

Depending on the concentration of these compounds, there might be adverse effects, as is the case with coumarin. Therefore, an upper limit was established for alcoholic beverages—10 mg/kg [53]—which was not reached in any treatment. A Tolerable Daily Intake (TDI) of 0.1 mg/kg bw has been established for coumarin [54]. Based on a 60 kg individual, a liter of cachaça needs to be consumed to reach the TDI.

Several of these types of phenolic compounds were also identified by Lima et al. [25] in commercial samples of cachaça aged in six different types of wood (peanut—*Pterogyne nitens*, garapa—*Apuleia leiocarpa*, freijo—*Cordia goeldiana*, umburana, and balsam—*Myroxylon peruiferum*). Amburana contained the highest levels of phenolics, whereas balsam had the least. According to these authors, catechin was the prevalent phenolic compound in peanut, freijo, and balsam, while it was piceatannol in garapa, coumarin in jatobá, and *trans*-cinnamic acid in amburana. Myricetin, which was the prevalent phenolic compound in jackfruit, was present at low levels. Therefore, myricetin could be an authenticity marker for jackfruit, which deserves further study. Studies are also needed to determine the impact of these compounds on the sensory characteristics of aged beverages.

### 3.8. Multivariate Analysis

**Figure 3:**
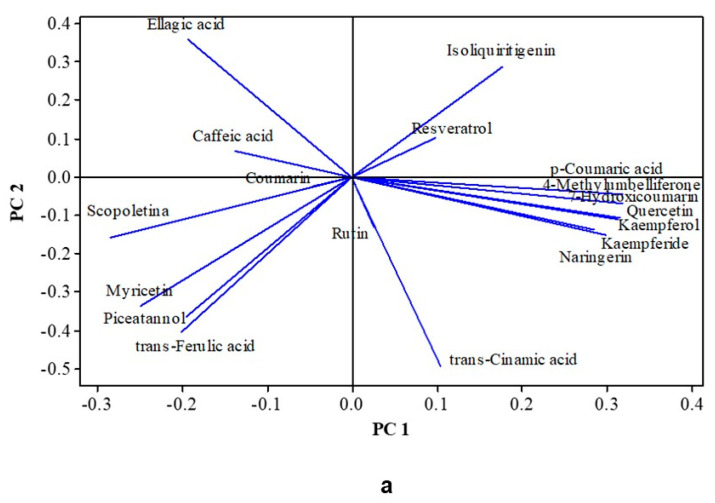

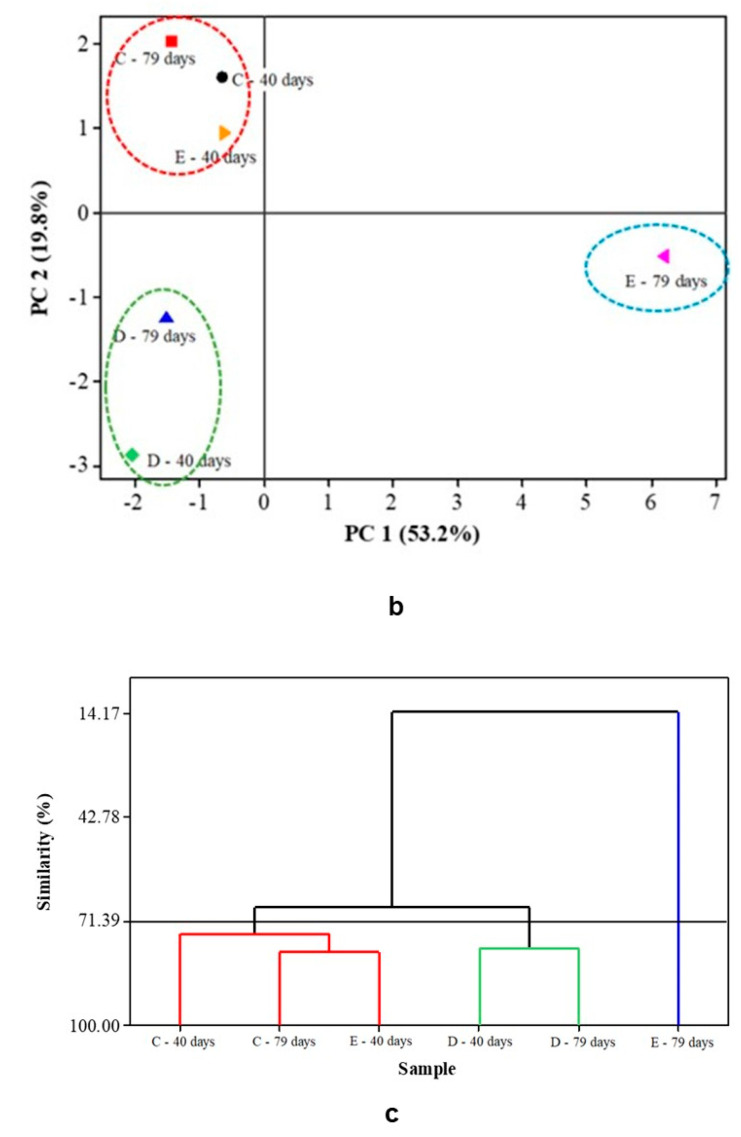
Principal Component Analyses (PCAs) and Hierarchical Cluster Analyses (HCAs) of phenolic compounds determined by HPLC in aged cachaça in jackfruit wood barrel or chip, under micro-aeration or not. (**a**) Loadings plot (PC1 + PC2 = 73.0%); (**b**) score plot showing clusters according to 70% similarity HCA; and (**c**) dendrogram obtained by clustering individual amine contents using average linkage and squared Pearson distance. Phenolic compounds (mg/L): coumarin, *trans*-cinnamic acid, caffeic acid, *p*-coumaric acid, ellagic acid, rutin, myricetin, quercetin, *iso*-liquiritigenin, kaempferol, kaempferide, naringerin, piceatannol, resveratrol, 7-hydroxycoumarin, scopoletin, *trans*-ferulic acid, and 4-methyl-umbelliferone. Each colored group (circle) in (**b**) corresponds to a cluster in (**c**).

Multivariate analyses of the auto-scaled data indicated that a two-principal components (PC) model explained 73.0% of the variance, e.g., PC1 explained 53.2% and PC2 19.8% of the variance (Figure 3). According to the PC loadings (Figure 3a) and the positions of the samples in the quadrants (Figure 3b), PC1 was affected, in a positive way, mainly by quercetin, kaempferol, kaempferide, 7-hydroxycoumarin, naringerin, *p*-coumaric acid, and 4-methyl-umbelliferone, and in a negative way by scopoletin and myricetin. PC2 was affected mainly by ellagic acid and *iso*-liquiritigenin in a positive way and by *trans*-cinnamic acid, *trans*-ferulic acid, and piceatannol in a negative way. HCA, as described in Figure 3c, separated the treatments, with 71% similarity, into three clusters. E-79 (chips used along with micro-aeration) was the only one on the positive side of PC1 and was characterized by the higher levels of quercetin, kaempferol, kaempferide, 7-hydroxycoumarin naringerin, *p*-coumaric acid, and 4-methyl-umbelliferone. The other two clusters were mainly associated with PC2, and they differed mainly due to the higher levels of ellagic acid and caffeic acid in the samples treated with jackfruit wood barrel (both 40 and 79 days aging) or the wood chips for a shorter time (40 days aging).

## 4. Conclusions

Using jackfruit wood, in aging the cachaça, both barrel and chips, caused significant changes in the parameters of quality studied. During aging, there were significant decreases in the alcoholic degree, copper, and lightness (L*), and increases in density, total and volatile acidity, total esters, dry extract, total sugars, and CIE colors (a*, b*, c*, and h). Increased aging time led to lower alcoholic degrees and total esters, whereas micro-aeration further decreased alcoholic degrees and total esters. No significant changes were observed regarding alcohols and higher alcohols. These changes are relevant as they provide a softer beverage with reduced residual copper from the distillation in copper stills.

The cachaças aged in jackfruit wood barrels for both periods were grouped with those aged with the chips without aeration for 40 days, showing the similarity of drinks aged in wood barrels and with wood chips for a shorter time. The other group showed a similarity for the cachaças aged with chips without aeration for 79 days and those with chips and aerated for 40 and 79 days. Myricetin was the prevalent phenolic compound in the aged cachaças. Since it is not common in other types of wood used for cachaça aging, it could be used as an authenticity marker for jackfruit wood-aged cachaça. This is a topic that deserves further study. The aged cachaça with wood chips, under aeration for 79 days, stood alone, characterized by the prevalence of several flavonoids and coumarins.

The use of jackfruit wood barrels during cachaça aging contributed to the incorporation of relevant phenolic compounds. The use of jackfruit chips provided a different phenolic’ profile irrespective of the aging time. When using chips and 40 days of aeration, it was possible to obtain a phenolic profile similar to that of jackfruit barrels. However, longer aging with chips under aeration led to a loss of phenolic compounds. So, to simulate the use of jackfruit barrels, aging under aeration for a shorter time is indicated.

Therefore, the use of jackfruit wood barrels or chips, micro-aeration, and aging affected the profile and levels of phenolic compounds, and this knowledge can be used to modulate the profile of phenolic compounds in the cachaças, enhancing desirable flavoring and health-promoting phenolics. Further studies are needed to investigate the impact of these health-promoting jackfruit wood phenolic compounds on the flavor of the aged cachaças.

Therefore, using jackfruit chips for cachaça aging can bring innovation to the sector, adding value to the final product, envisioning both national and international markets.

## Figures and Tables

**Table 1:** Physicochemical characteristics and inorganic compounds of *cachaça* submitted to different treatments—not aged (Control), aged in stainless steel (or jackfruit barrels in the presence or not of jackfruit wood chips, under micro-aeration or not (A–E) for 40 and 79 days (40, 79).

Sample/Aging Time (days)	Alcoholic Degree (% *v/v*)	Density at 20 °C (g/L)	Total Acidity †	Volatile Acidity †	Total Esters ‡	Dry Extract (g/L)	Total Sugars (g/L)	Cu (mg/L)	Zn (mg/L)
Control	48.30 ± 0.01 a	0.935 ± 0.001 e	16.95 ± 0.00 e	32.39 ± 1.81 e	27.5 ± 0.55 d	0.04 ± 0.06 e	0.17 ± 0.01 c	1.17 ± 0.06 a	0.11 ± 0.01 c
A—40	47.00 ± 0.06 ab	0.938 ± 0.001 d	16.95 ± 0.00 e	32.21 ± 0.04 e	27.4 ± 0.84 d	0.04 ± 0.01 e	0.17 ± 0.01 c	1.07 ± 0.06 a	0.12 ± 0.01 abc
A—79	48.17 ± 1.17 a	0.940 ± 0.001 c	17.22 ± 0.00 e	33.38 ± 0.11 e	27.4 ± 0.09 d	0.04 ± 0.01 e	0.20 ± 0.04 c	1.20 ± 0.17 a	0.12 ± 0.01 bc
B—40	46.01 ± 0.30 bc	0.939 ± 0.001 c	16.95 ± 0.25 e	37.29 ± 2.00 cde	32.5 ± 2.05 cd	0.03 ± 0.01 e	0.18 ± 0.01 c	1.20 ± 0.01 a	0.16 ± 0.04 a
B—79	43.76 ± 0.66 ef	0.944 ± 0.001 b	17.90 ± 0.59 e	39.47 ± 1.93 bcd	36.5 ± 1.56 bc	0.04 ± 0.01 e	0.21 ± 0.02 c	1.23 ± 0.06 a	0.15 ± 0.01 ab
C—40	45.71 ± 0.57 bc	0.941 ± 0.001 c	68.63 ± 3.49 d	42.31 ± 2.02 abc	39.3 ± 2.20 b	5.48 ± 0.99 d	0.18 ± 0.01 c	0.62 ± 0.03 b	nd
C—79	45.24 ± 0.64 cde	0.943 ± 0.001 b	112.1 ± 13.7 bc	46.06 ± 2.01 a	42.6 ± 1.23 b	7.45 ± 1.23 c	0.49 ± 0.08 b	0.53 ± 0.05 b	nd
D—40	45.48 ± 0.11 bcd	0.940 ± 0.001 c	96.88 ± 6.06 c	43.39 ± 0.11 ab	54.9 ± 1.90 a	8.40 ± 0.69 bc	0.56 ± 0.01 ab	0.73 ± 0.04 b	nd
D—79	45.21 ± 0.65 cde	0.944 ± 0.001 b	129.6 ± 12.80 b	46.70 ± 2.23 a	32.5 ± 2.26 cd	9.78 ± 0.87 ab	0.61 ± 0.02 a	0.63 ± 0.09 b	nd
E—40	43.93 ± 0.31 de	0.943 ± 0.001 b	107.0 ± 3.49 c	35.61 ± 1.94 de	52.9 ± 2.35 a	9.08 ± 0.12 bc	0.57 ± 0.02 ab	0.71 ± 0.05 b	nd
E—79	42.24 ± 0.49 f	0.948 ± 0.001 a	158.1 ± 8.11 a	46.90 ± 2.34 a	32.5 ± 5.36 cd	10.81 ± 0.13 a	0.59 ± 0.02 a	0.65 ± 0.10 b	nd

† Total and volatile acidity expressed in mg acetic acid/100 mL. ‡ Total esters expressed in mg ethyl acetate/100 mL anhydrous alcohol. Control (not aged); A—aged in stainless steel tanks; B—aged in stainless steel tanks under micro-aeration; C—aged in jackfruit barrel; D—aged in stainless steel tanks with jackfruit chips; E—aged in stainless steel tanks with jackfruit chips under micro-aeration. The aging times were 40 and 79 days. nd: not detected. Mean values ± standard deviation with different letters in the same column are significantly different (Tukey’s test, *p* < 0.05).

**Table 2:** Levels of alcohols and total higher alcohols in cachaças submitted to different treatments—not aged (control), aged in stainless steel or jackfruit barrels in the presence or not of jackfruit wood chips, aerated or not during 40 and 79 days.

Sample/Aging	Level (mg/100 mL Anhydrous Alcohol)			
Time (Days)	Isobutyl Alcohol	Isoamyl Alcohol	Propyl Alcohol	∑ Higher Alcohols
Control	5.62 ± 0.04 a	9.31 ± 0.03 ab	8.97 ± 0.12 c	23.9 ± 0.00 ab
A—40	4.37 ± 0.18 bc	8.98 ± 0.83 ab	10.4 ± 0.41 abc	23.8 ± 1.38 ab
A—79	3.78 ± 0.20 c	8.18 ± 1.19 ab	9.42 ± 0.66 bc	21.4 ± 2.05 b
B—40	4.26 ± 0.34 bc	9.49 ± 0.90 ab	10.2 ± 0.79 abc	24.0 ± 2.02 ab
B—79	4.69 ± 0.26 bc	10.6 ± 0.99 ab	11.4 ± 0.59 ab	26.6 ± 1.74 ab
C—40	4.53 ± 0.15 bc	9.25 ± 0.26 ab	10.9 ± 0.50 abc	24.7 ± 0.90 ab
C—79	4.14 ± 0.16 bc	7.64 ± 0.91 b	9.89 ± 0.35 bc	21.7 ± 1.42 b
D—40	4.56 ± 0.61 bc	9.54 ± 1.57 ab	10.9 ± 1.30 abc	25.0 ± 3.47 ab
D—79	4.6 ± 0.38 bc	10.2 ± 0.72 ab	11.0 ± 0.89 abc	25.7 ± 1.98 ab
E—40	5.02 ± 0.25 ab	10.8 ± 0.66 a	12.3 ± 0.84 a	28.2 ± 1.57 a
E—79	4.78 ± 0.47 ab	10.2 ± 1.52 a	11.5 ± 1.08 ab	26.5 ± 3.06 ab

Control (not aged); A—aged in stainless steel barrels; B—aged in stainless steel barrels under micro-aeration; C—aged in jackfruit barrels; D—aged in stainless steel barrels with jackfruit chips; E—aged in stainless steel barrels with jackfruit chips under micro-aeration. The aging times were 40 and 79 days. n-Butyl alcohol, 2-butyl alcohol, and methyl alcohol were not detected in all treatments. Mean values ± standard deviation with different letters in the same column are significantly different (Tukey’s test, *p* < 0.05).

**Table 3:** CIE L*a*b* color characteristics of *cachaça* submitted to different treatments—not aged (Control), aged in stainless steel or jackfruit barrels in the presence or not of jackfruit wood chips, micro-aerated or not (A–E) for 40 and 79 days.

Sample/Aging	CIE L*a*b* Color Characteristics				
Time (Days)	L*	a*	b*	C*	h
Control	96.6 ± 0.01 a	−0.28 ± 0.06 e	1.50 ± 0.02 c	1.52 ± 0.02 c	100.8 ± 0.12 b
A—40	103 ± 0.09 a	−0.55 ± 0.01 e	−0.14 ± 0.06 c	0.57 ± 0.01 c	197.3 ± 3.50 a
A—79	96.7 ± 0.02 a	−0.09 ± 0.29 e	1.40 ± 0.05 c	1.44 ± 0.07 c	100.4 ± 0.10 b
B—40	103 ± 0.06 a	−0.46 ± 0.05 e	−0.18 ± 0.05 c	0.50 ± 0.04 c	200.8 ± 4.78 a
B—79	96.5 ± 0.11 a	−0.25 ± 0.04 e	1.58 ± 0.23 c	1.60 ± 0.23 c	99.07 ± 0.42 b
C—40	81.6 ± 2.27 b	11.7 ± 2.59 d	59.8 ± 9.53 b	60.7 ± 9.65 b	79.01 ± 0.88 c
C—79	75.3 ± 1.86 bc	21.4 ± 1.89 cd	74.9 ± 1.48 a	78.0 ± 1.04 a	74.04 ± 1.61 cd
D—40	68.0 ± 6.30 cd	28.9 ± 8.51 abc	61.2 ± 6.47 ab	68.2 ± 3.40 ab	64.55 ± 8.43 de
D—79	61.4 ± 7.20 d	35.6 ± 7.29 a	56.7 ± 11.2 b	67.7 ± 6.12 ab	57.32 ± 9.87 e
E—40	70.1 ± 0.37 cd	25.1 ± 0.74 bc	64.4 ± 1.1 ab	69.1 ± 1.29 ab	68.70 ± 0.24 cde
E—79	63.9 ± 0.40 d	33.6 ± 0.16 ab	62.8 ± 0.95 ab	71.2 ± 0.91 ab	61.86 ± 0.26 de

Control (not aged); A—aged in stainless steel barrels; B—aged in stainless steel barrels under micro-aeration; C—aged in jackfruit barrels; D—aged in stainless steel barrels with jackfruit chips; E—aged in stainless steel barrels with jackfruit chips under micro-aeration. The aging times were 40 and 79 days. Mean values ± standard deviation with different letters in the same column are significantly different (Tukey’s test, *p* < 0.05).

**Table 4:** Levels of phenolic compounds and coumarins determined by HPLC-DAD-FLD and total phenolic content (Folin–Ciocalteu—FC) in *cachaça* submitted to different treatments—aged in stainless steel or jackfruit barrels in the presence or not of jackfruit wood chips, aerated or not (A–E) for 40 and 79 days (40, 79).

Phenolic Compounds	Mean Levels ± Standard Deviation (mg/L)					
	C—40	C—79	D—40	D—79	E—40	E—79
*Phenolic acids*						
Caffeic acid	3.33 ± 1.99 bc	2.57 ± 0.98 bc	3.93 ± 2.61 b	3.11 ± 0.61 bc	8.42 ± 2.15 a	1.18 ± 0.01 bc
*trans*-Cinnamic acid	0.76 ± 0.02 b	0.83 ± 0.47 ab	1.46 ± 0.40 a	1.25 ± 0.89 a	0.92 ± 0.18 ab	1.38 ± 0.06 a
*p*-Coumaric acid	0.56 ± 0.46 b	0.87 ± 0.91 b	0.31 ± 0.20 b	0.63 ± 0.52 b	0.48 ± 0.05 b	7.43 ± 0.08 a
Ellagic acid	2.20 ± 2.18 a	3.55 ± 4.13 a	0.88 ± 1.06 a	2.49 ± 2.33 a	2.31 ± 0.36 a	0.48 ± 0.06 a
*trans*-Ferulic acid	3.94 ± 1.98 a	1.61 ± 1.11 a	12.0 ± 8.67 a	8.39 ± 11.7 a	3.65 ± 0.47 a	0.25 ± 0.01 a
*Stilbene*						
Piceatannol	1.48 ± 0.75 a	2.43 ± 2.48 a	4.44 ± 3.46 a	3.01 ± 4.08 a	1.17 ± 0.11 a	1.03 ± 0.03 a
Resveratrol	2.03 ± 0.92 a	0.80 ± 0.20 abc	1.17 ± 0.80 abc	0.64 ± 0.47 bc	0.90 ± 0.51 abc	1.40 ± 0.20 ab
*Flavonoids*						
*iso*-Liquiritigenin	0.77 ± 0.92 b	2.13 ± 0.57 a	0.19 ± 0.01 b	0.28 ± 0.13 b	0.55 ± 0.54 b	1.89 ± 0.15 a
Kaempferol	1.08 ± 0.11 a	0.92 ± 0.06 a	1.18 ± 0.30 a	1.03 ± 0.45 a	1.28 ± 0.26 a	2.26 ± 3.51 a
Kaempferide	0.19 ± 0.02 b	0.33 ± 0.18 b	0.57 ± 0.29 b	0.59 ± 0.40 b	0.94 ± 1.02 ab	1.74 ± 0.04 a
Myricetin	6.32 ± 3.06 bc	7.14 ± 5.25 bc	18.2 ± 1.95 a	14.7 ± 9.21 ab	7.46 ± 1.01 bc	0.15 ± 0.02 c
Naringerin	0.76 ± 0.04 b	0.59 ± 0.05 b	0.78 ± 0.14 b	0.72 ± 0.33 b	0.81 ± 0.03 b	1.16 ± 0.05 a
Quercetin	0.44 ± 0.08 b	0.43 ± 0.15 b	0.79 ± 0.13 b	1.10 ± 1.14 b	1.14 ± 0.30 b	5.79 ± 0.55 a
Rutin	1.22 ± 1.56 a	0.46 ± 0.66 a	0.06 ± 0.15 a	5.16 ± 8.81 a	0.18 ± 0.04 a	1.89 ± 0.13 a
*Coumarins*						
Coumarin	0.31 ± 0.40 a	1.42 ± 1.21 a	1.23 ± 0.07 a	0.78 ± 0.77 a	1.43 ± 0.80 a	0.93 ± 0.01 a
4-Methyl-umbelliferone	0.01 ± 0.01 b	0.01 ± 0.01 b	0.02 ± 0.01 b	0.01 ± 0.01 b	0.01 ± 0.01 b	3.80 ± 0.28 a
7-Hydroxi-coumarin	0.02 ± 0.01 b	0.01 ± 0.01 b	0.01 ± 0.01 b	0.00 ± 0.01 b	0.01 ± 0.01 b	1.28 ± 0.40 a
Scopoletin	0.23 ± 0.04 ab	0.33 ± 0.23 ab	0.47 ± 0.27 a	0.27 ± 0.34 ab	0.24 ± 0.05 ab	0.02 ± 0.01 b
*Total phenolic-FC*	228.1 ± 17.29 d	370.3 ± 48.27 abc	292.5 ± 58.44 cd	393.3 ± 65.69 ab	301.5 ± 38.73 bc	426.8 ± 10.76 a

C—aging in jackfruit barrel; D—aging in stainless steel barrels with jackfruit chips; E—aging in stainless steel barrels with jackfruit chips under micro-aeration. The aging times were 40 and 79 days. Control, A, and B treatments—no phenolic compound was detected. Mean values ± standard deviation with different letters in the same column are significantly different (Tukey’s test, *p* < 0.05). To statistically evaluate possible correlations among parameters, a Pearson analysis was applied. There was a significant positive correlation between TPC and the color parameters, but there was color saturation (C* value) in the treatments C, D, and E after 40 days of aging. Another positive and significant correlation was observed for TPC and dry extract (*r* = 0.829, *p* = 0.042). Dry extract and lightness correlated significantly (*r* =−0.886, *p* = 0.019). And, finally, there was a significant correlation (*r* = 0.886, *p* = 0.019) between dry extract and the color parameter a*.

## Data Availability

The original contributions presented in the study are included in the article. Further inquiries can be directed to the corresponding author.

## References

[1] Ratkovich N., Esser C., de Resende Machado A.M., Mendes B.d.A., Cardoso M.d.G. (2023). The Spirit of Cachaça Production: An Umbrella Review of Processes, Flavour, Contaminants and Quality Improvement. Foods.

[2] Barbosa R.B., Santiago W.D., Alvarenga G.F., Oliveira R.E.S., Ferreira V.R.F., Nelson D.L., Cardoso M.G. (2022). Physical–chemical profile and quantification of phenolic compounds and polycyclic aromatic hydrocarbons in cachaça samples aged in oak (*Quercus* sp.) barrels with different heat treatments. Food Bioprocess. Technol..

[3] Brasil Ministério da Agricultura, Pecuária e Abastecimento (MAPA). Portaria nº 539, de 26 de Dezembro 2022. https://www.in.gov.br/en/web/dou/-/portaria-mapa-n-539-de-26-de-dezembro-de-2022-453828778.

[4] IBRAC (2022). Instituto Brasileiro da Cachaça. Mercado Interno. https://ibrac.net/servicos/mercado-interno.

[5] Brasil Ministério da Agricultura, Pecuária e Abastecimento (MAPA). Portaria nº 90, de 23 de Agosto de 2016. https://www.gov.br/agricultura/pt-br/acesso-a-informacao/participacao-social/consultas-publicas/documentos/portaria-90-2016-envelhecimento-de-bebidas.pdf.

[6] Maia A.B., Marinho L.S., Tonidandel L.O., Conceição E.C., Bárbara Dias Machado B.D. (2023). Wood chips and cachaça aging. Int. J. Dev. Res..

[7] Cardoso M.G., Machado A.M.R., Caetano A.R.S., Campolina G.A., Nelson D.L., Reis F.R., dos Santos C.M.E. (2022). Volatile compounds formation in cachaça. Volatile Compounds Formation in Specialty Beverages.

[8] Castro M.C., Silvello G.C., Corniani L.S., Acevedo M.S.M.S.F., Pereira A.A.M., Alcarde A.R. (2023). Maturation-related phenolic compounds in cachaça aged in oak barrels: Influence of reuses. Wood Sci. Technol..

[9] Rodrigues L.M.A., Cardoso M.G., Santiago W.D., Barbosa R.B., Santiago J.D.A., Lima L.M.Z., Nelson D.L. (2020). Organic contaminants in distilled sugar cane spirits produced by column and copper alembic distillation. Res. Soc. Dev..

[10] Feng Z., Martínez-Lapuente L., Palacios A., Ayestarán B., Guadalupe Z. (2024). Influence of *Quercus alba* oak geographical origin on the colour characteristics and phenolic composition of Tempranillo wines. Eur. Food Res. Technol..

[11] Silva F.A., Morais K.C.R.C., Ribeiro K.O., Garcia L.G.C., Caliari M. (2020). Evolution of the content of phenolic compounds, antioxidant activity and color in organic sugarcane spirit aged in barrels of different woods. Res. Soc. Dev..

[12] Jordão A.J., Correia A.C., Botelho R.V., Ortega-Heras M., González-San José M.L. (2023). Potential of the enological use of several Brazilian wood species on the phenolic composition and sensory quality of different wines. BIO Web Conf..

[13] Bortoletto A.M., Correa A.C., Alcarde A.R. (2016). Aging practices influence chemical and sensory quality of cachaça. Food Res. Int..

[14] Canas S., Caldeira I., Anjos O., Belchior A.P. (2019). Phenolic profile and colour acquired by the wine spirit in the beginning of ageing: Alternative technology using micro-oxygenation vs. traditional technology. LWT—Food Sci. Technol..

[15] Sanches-Gómez R., Del Álamo-Sanza M., Nevares I. (2020). Volatile composition of oak wood from different customized oxygenation wine barrels: Effect on red wine. Food Chem..

[16] Wang L., Chen S., Xu Y. (2022). Distilled beverage aging: A review on aroma characteristics, maturation mechanisms, and artificial aging techniques. Compr. Rev. Food Sci. Food Saf..

[17] Granja-Soares J., Roque R., Cabrita M.J., Anjos O., Belchior A.P., Caldeira I., Canas S. (2020). Effect of innovative technology using staves and micro-oxygenation on the odorant and sensory profile of aged wine spirit. Food Chem..

[18] Caldeira I., Vitória C., Anjos O., Fernandes T.A., Gallardo E., Fargeton L., Boissier B., Catarino S., Canas S. (2021). Wine spirit ageing with chestnut staves under different micro-oxygenation strategies: Effects on the volatile compounds and sensory profile. Appl. Sci..

[19] Gollihue J., Pook V.G., DeBolt S. (2021). Sources of variation in bourbon whiskey barrels: A review. J. Inst. Brew..

[20] Mahanta C.L., Kalita D., Preedy V. (2015). Chapter 47—Jackfruit processing and utilization of jackfruit seeds. Processing and Impact on Active Components in Food.

[21] Castro M.C., Bortoletto A.M., Silvello G.C., Alcarde A.R. (2020). Lignin-derived phenolic compounds in cachaça aged in new barrels made from two oak species. Heliyon.

[22] Brasil. Ministério da Agricultura, Pecuária e do Abastecimento (2005). Instrução Normativa nº 13, de 29 de Junho de 2005. Aprova o Regulamento Técnico para Fixação dos Padrões de Identidade e Qualidade para Aguardente de Cana e para Cachaça. Diário Oficial da União.

[23] Amerine M.A., Ough C.S. (1980). Methods for Analysis of Musts and Wines.

[24] Saito M.S., Santos W.A., Mamede M.E.O. (2024). Coffee flavoured kombucha: Development, physicochemical characterization and sensory analysis. Fermentation.

[25] Lima D.A.R., Alves E.C., Anjos J.P. (2024). Greener method proposal for the determination of phenolic compounds and coumarins in aged sugarcane spirits using green bio-based solvents in HPLC-DAD-FLD system. Green. Anal. Chem..

[26] Silvello G.C., Bortoletto A.M., Castro M.C., Alcarde A.R. (2021). New approach for barrel-aged distillates classification based on maturation level and machine learning: A study of cachaça. LWT—Food Sci. Technol..

[27] Morais K.C.R.C., Jesus L.S., Ribeiro G.O., Caliari M., Silva F.A., Lião L.M. (2022). Chemical evaluation of cachaça aged in different Brazilian woods. Beb Ferment. Destil Pesq. Aplic..

[28] García-Moreno M.V., Sánchez-Guillén M.M., Delgado-González M.J., Durán-Guerrero E., Rodríguez-Dodero M.C., García-Barroso C., Guillén-Sánchez D.A. (2021). Chemical content and sensory changes of oloroso sherry wine when aged with four different wood types. LWT—Food Sci. Technol..

[29] Santiago W.D., Cardoso M.G., Nelson D.L. (2017). Cachaça stored in casks newly constructed of oak (*Quercus* sp.), amburana (*Amburana cearensis*), jatoba (*Hymenaeae carbouril*), balsam (*Myroxylon peruiferum*) and peroba (*Paratecoma peroba*): Alcohol content, phenol composition, colour intensity and dry extract. J. Inst. Brew..

[30] Biagioni M.A. (2021). Papel da madeira no envelhecimento da cachaça. Rev. Cient. Multidiscip..

[31] Zacaroni L.M., Cardoso M.G., Santiago W.D., Mendonça J.G.P., Nunes C.A., Duarte F.C. (2014). Avaliação multivariada de composição fenólica de cachaças envelhecidas em diferentes barris de madeira. Científica.

[32] ATSDR (2023). Agency for Toxic Substances and Disease Registry. Toxicological Profile for Copper (Draft for Public Comment).

[33] Clark A.C., Wilkes E.N., Scollary G.R. (2015). Copper in white wine. Aust. J. Grape Wine Res..

[34] ATSDR (2021). Agency for Toxic Substances and Disease Registry. Toxicological Profile for Zinc.

[35] ATSDR (2015). Agency for Toxic Substances and Disease Registry. Toxicological Profile for Aluminum.

[36] Bortoletto A.M., Hill A., Jack F. (2023). Rum and cachaça. Distilled Spirits.

[37] Lima C.M.G., Benoso P., Pierezan M.D., Santana R.F., Hassemer G.S., Rocha R.A., Nora F.M.D., Verruck S., Caetano D., Simal-Gandara J. (2022). A state-of-the-art review of the chemical composition of sugarcane spirits and current advances in quality control. J. Food Compos. Anal..

[38] Nekoukar Z., Zakariaei Z., Taghizadeh F., Musavi F., Banimostafavi E.S., Sharifpour A., Ebrahim Ghuchi N., Fakhar M., Tabaripour R., Safanavaei S. (2021). Methanol poisoning as a new world challenge: A review. Ann. Med. Surg..

[39] Carvalho D.G., Ranzan L., Trierweiler L.F., Trierweiler J.O. (2020). Determination of the concentration of total phenolic compounds in aged cachaça using two-dimensional fluorescence and mid-infrared spectroscopy. Food Chem..

[40] Zhang N., Yang Y., Wang X., Shi T., Lv P., Li Q.X., Hua R. (2024). Myricetin inhibits photodegradation of profenofos in water: Pathways and mechanisms. Agronomy.

[41] Chavez-Santiago J.O., Rodríguez-Castillejos G.C., Montenegro G., Bridi R., Valdés-Gómez H., Alvarado-Reyna S., Castillo-Ruiz O., Santiago-Adame R. (2022). Phenolic content, antioxidant, and antifungal activity of jackfruit extracts (*Artocarpus heterophyllus* Lam.). Food Sci. Technol..

[42] Semwal D.K., Semwal R.B., Combrinck S., Viljoen A. (2016). Myricetin: A Dietary Molecule with Diverse Biological Activities. Nutrients.

[43] Imran M., Saeed F., Hussain G., Imran A., Mehmood Z., Gondal T.A., El-Ghorab A., Ahmad I., Pezzani R., Arshad M.U. (2021). Myricetin: A comprehensive review on its biological potentials. Food Sci. Nutri..

[44] Rezaeiroshan A., Saeedi M., Morteza-Semnani K., Akbari J., Hedayatizadeh-Omran A., Goli H., Nokhodchi A. (2022). Vesicular formation of *trans*-ferulic acid: An efficient approach to improve the radical scavenging and antimicrobial properties. J. Pharm. Innov..

[45] Bhuia M.S., Rokonuzzman M., Hossain M.I., Ansari S.A., Ansari I.A., Islam T., Al Hasan M.S., Mubarak M.S., Islam M.T. (2023). Anxiolytic-like effects by *trans*-ferulic acid possibly occur through GABAergic interaction pathways. Pharmaceuticals.

[46] Mude H., Balapure A., Thakur A., Ganesan R., Ray Dutta J. (2022). Enhanced antibacterial, antioxidant and anticancer activity of caffeic acid by simple acid-base complexation with spermine/spermidine. Nat. Prod. Res..

[47] Cizmarova B., Hubkova B., Bolerazska B., Marekova M., Birkova A. (2020). Caffeic acid: A brief overview of its presence, metabolism, and bioactivity. Bioact. Compd. Health Dis..

[48] Golmei P., Kasna S., Roy K.P., Kumar S. (2024). A review on pharmacological advancement of ellagic acid. J. Pharmacol. Pharmacother..

[49] Carrillo-Martinez E.J., Flores-Hernández F.Y., Salazar-Montes A.M., Nario-Chaidez H.F., Hernández-Ortega L.D. (2024). Quercetin, a flavonoid with great pharmacological capacity. Molecules.

[50] Pibuel M.A., Poodts D., Sias S.A., Byrne A., Hajos S.E., Franco P.G., Lompardía S.L. (2023). 4-Methylumbelliferone enhances the effects of chemotherapy on both temozolomide-sensitive and resistant glioblastoma cells. Sci. Rep..

[51] Al-Jaber H.I., Shakya A.K., Al-Qudah M.A., Barhoumi L.M., Abu-Sal H.E., Hasan H.S., Al-Bataineh N., Abu-Orabi S., Mubarak M.S. (2024). Piceatannol, a comprehensive review of health perspectives and pharmacological aspects. Arab. J. Chemi..

[52] Rauf A., Khalil A.A., Awadallah S., Khan S.A., Abu-Izneid T., Kamran M., Hemeg H.A., Mubarak M.S., Khalid A., Wilairatana P. (2024). Reactive oxygen species in biological systems, pathways, associated diseases, and potential inhibitors: A review. Food Sci. Nutri..

[53] Codex Alimentarius (2008). Codex Alimentarius: General Requirements for Natural Flavourings (CAC/GL 66-2008). https://www.fao.org/fao-who-codexalimentarius/sh-proxy/de/?lnk=1&url=https%253A%252F%252Fworkspace.fao.org%252Fsites%252Fcodex%252FMeetings%252FCX-714-44%252FCRDs%252Ffl44_crd02x.pdf.

[54] AFC (2004). Opinion of the scientific panel on food additives, flavourings, processing aids and materials in contact with food (AFC) on a request from the Commission related to coumarin. EFSA J..

